# Challenges and Effects of the COVID-19 Pandemic on Asylum Seeker Health at the U.S.-Mexico Border

**DOI:** 10.1089/heq.2020.0110

**Published:** 2021-04-13

**Authors:** Christopher W. Reynolds, Vidya Ramanathan, Elena Lorenzana, Porag J. Das, Kyra M. Sagal, Kristen M. Lozada-Soto, Linda Camaj Deda, Anisa S. Haque, Florian F. Schmitzberger, Grecia Quiroga, Sarah A. Raven, Michele Heisler

**Affiliations:** ^1^ University of Michigan Medical School, Ann Arbor, Michigan, USA.; ^2^ University of Michigan Asylum Collaborative, Ann Arbor, Michigan, USA.; ^3^ Department of Pediatrics, University of Michigan, Ann Arbor, Michigan, USA.; ^4^ Department of Emergency Medicine, University of Michigan, Ann Arbor, Michigan, USA.; ^5^ Physicians for Human Rights, Ann Arbor, Michigan, USA.; ^6^ Department of Internal Medicine, University of Michigan, Ann Arbor, Michigan, USA.

**Keywords:** asylum health care, COVID-19, immigrant health, health care services delivery, global health, U.S.-Mexico border

## Abstract

**Purpose:** The coronavirus disease 2019 (COVID-19) pandemic presents health care challenges to asylum seekers living in congregate encampments, including those along the U.S.-Mexico border. It is necessary to understand the impact of the pandemic among this population to address health care needs, reduce transmission, and diminish COVID-19-related morbidity.

**Methods:** Thirty interviews were conducted with asylum seekers and health care professionals in a temporary camp in Matamoros, Mexico to determine challenges, perceptions, and effects of the COVID-19 pandemic. Interviews were coded in NVivo12 by using a team-based approach.

**Results:** The pandemic caused significant mental health burdens but no perceived adverse physical effects, with the U.S. border closure and health care access barriers as more pressing concerns. Participants reported access to information about COVID-19 but had varied levels of knowledge and adherence to disease reduction strategies due to camp conditions. Most participants believed that they had special protection from COVID-19, including strong immune systems or from God. The nongovernmental organizations providing health care and sanitation faced multiple challenges to implement new policies to manage COVID-19. The institution of required temperature checks and quarantine of COVID-19 positive patients led to distrust, decreased seeking of health care services among asylum seekers, and possible underreporting of COVID-19 cases.

**Conclusion:** Our findings among asylum seekers in a Matamoros camp highlight the challenges to implementing disease reduction policies in low-resource congregate camps. Policies to address disease outbreaks focusing on the social determinants of health, health care access barriers, and community engagement may be more acceptable to asylum seekers, suggesting the need for effective strategies to provide prevention information that complements such measures.

## Introduction

Severe Acute Respiratory Syndrome Coronavirus-2, causing coronavirus disease 2019 (COVID-19) disease, has had a devastating impact, with nearly two million deaths worldwide.^1^ COVID-19 disproportionately affects vulnerable populations, including refugees and asylum seekers.^2–5^ Although social distancing, hand hygiene, and mask wearing are recommended to reduce transmission,^6^ these strategies are difficult in refugee camps due to close living and decreased sanitation.^7^ Many camps report a lack of information, varied compliance with public health measures, and insufficient testing, leading to inconsistent knowledge and underreporting of cases.^8–10^ Though little information exists to describe COVID-19 among asylum seekers in congregate settings, disease outbreaks historically presented challenges to this population. One study demonstrated that 108 refugee camps were affected by 364 outbreaks from 2009 to 2017, showing high infection rates and inconsistent compliance with disease reduction strategies.^11–14^ A study analyzing the potential effects of COVID-19 on high-density refugee populations showed that a large-scale outbreak is likely and could have devastating effects.^15^

After the U.S. government's implementation of the Migrant Protection Protocols (MPP) in January 2019, thousands of asylum seekers established a tent encampment in Matamoros, Mexico, on the U.S.-Mexico border. The MPP forced asylum seekers to remain in Mexico while their claims for asylum in the United States were adjudicated, rather than in the United States as is called for according to asylum law.^16,17^ The COVID-19 pandemic further impacted asylum seekers, as asylum court proceedings were shut down.^18^ Before the pandemic, asylum seekers in Matamoros faced significant health care needs and access barriers and relied on nongovernmental organizations (NGOs) for medical care.^19^ Global Response Management, one such organization, reported in July 2020 increased levels of malnutrition, dehydration, respiratory illness, and gastrointestinal diseases among this population.^20^ These conditions raise significant concerns about new challenges in the face of a COVID-19 outbreak.

It is necessary to understand the impact of COVID-19 on asylum seekers in the camps at the U.S.-Mexico border to implement feasible disease reduction strategies and improve health care outcomes for this vulnerable population. Such findings could help to fill the knowledge gap of how COVID-19 impacts low-resource settings. This study aimed at describing the effects, perceptions, and challenges of COVID-19 on the asylum seeker camp in Matamoros, Mexico by soliciting the perspectives of asylum seekers and health care professionals working with them.

## Methods

### Study design

A semi-structured interview was adapted from qualitative studies among asylum, refugee, and ex-combatant populations in Latin America.^21^ The script was content-validated with health care professionals and asylum seekers (*n*=6), with the final version lasting 45 min and including sections on COVID-19 challenges, perceptions, and policies (Appendices A1 and A2).

Inclusion criteria were asylum seekers and health care professionals in the Matamoros, Mexico camp. In August 2020, participants were recruited through a convenience snowball method, determined most appropriate for an exploratory study to achieve varied sample demographics. Interviews were conducted in-person in a private location within the camp, or at a nearby NGO office. Consent was obtained verbally before beginning each interview, which was audio recorded with the EasyVoiceRecorder (Digipom, Quebec, Canada) app. Recordings were collected on password protected cell phones, uploaded to a secure DropBox (San Francisco, CA) folder, and deleted from the cell phone. Data collection was completed once the research team determined that thematic saturation had occurred.^22^

### Data analysis

Study participants were assigned a unique code used throughout qualitative analysis to guarantee anonymity. All recorded interviews were transcribed in Spanish and stored on an encrypted device. Codebook development was conducted according to a qualitative team-based coding approach.^23^ After data immersion, a codebook was developed by two researchers and validated by using a repetitive process of increasing inter-coder agreement via independent transcription. After two rounds of coding, an inter-coder reliability of 0.63 using Cohen's Kappa (*κ*=0.63) was considered acceptable.^24^ Transcripts were coded in NVivo12 by six study investigators with the validated codebook. Binary questions were coded for frequency data and tabulated. Quotes reflecting major themes were translated into English and selected to include a range of participants. To ensure data reliability, the following designs were implemented: (1) source triangulation by interviewing asylum seekers and health care professionals, (2) thematic codebook verification using independent and cooperative techniques, and (3) consensus on final results agreed on by all research members. This study was approved by the University of Michigan IRBMED and adhered to COREQ guidelines.^25^

## Results

### Demographics

**FIG. 1.**
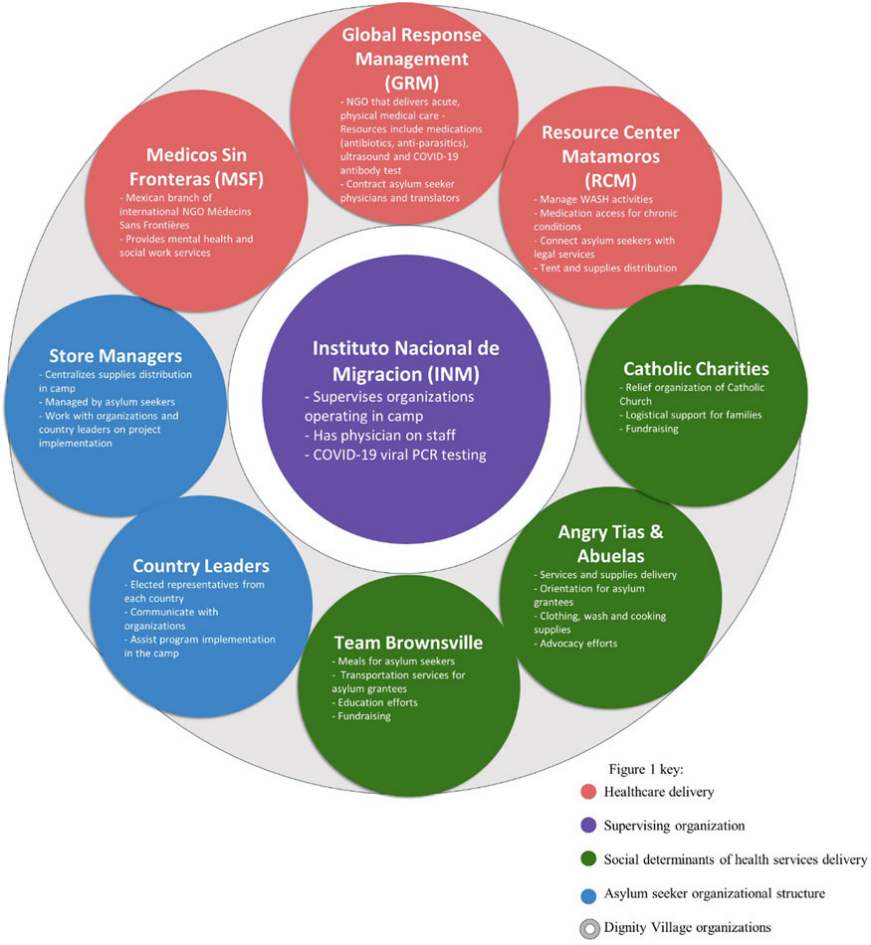
Organizational structure of Mexican immigration authorities, nongovernmental organizations, and asylum seeker community leaders of the Matamoros, Mexico asylum seeker camp. This figure describes the organizational structure of the various humanitarian organizations working in the asylum seeker camp of Matamoros, Mexico. The organizations are divided into colors by area of focus, but often collaborate across specialty area. Organizations within the grey ring comprise Dignity Village, which works with Instituto Nacional de Migración, the branch of the Mexican government that oversees immigration and border control.

Thirty interviews were conducted among 20 asylum seekers and 10 health care professionals in the Matamoros, Mexico asylum seeker camp (Tables 1 and 2). Four health care professionals were also asylum seekers, contracted by the medical NGO. Sixty-five percent of asylum seekers were unemployed and reported being unable to work to care for their children. Nearly all asylum seekers (90%) were unable to return home, citing border closures and safety fears. Among 10 health care professionals, 7 provided health care and 3 worked by delivering services. Various organizations addressed the humanitarian needs of asylum seekers, forming a collaborative network called Dignity Village to coordinate services delivery (Fig. 1).

**Table 1.** Demographics of Asylum Seekers in Matamoros, Mexico

	Age	Gender	Birthplace	Highest education	Work status	Time in camp	Came with family	Has children	Applied for asylum in the United States	Received asylum response	Would return home	Applied for asylum in another country
AS01	43	Female	El Salvador	Primary	Unemployed	10 months	Yes	Yes	Yes	No	No	No
AS02	24	Female	El Salvador	None	Unemployed	1 year	Yes	Yes	Yes	No	No	No
AS03	27	Female	El Salvador	Secondary	Informal	1 year	Yes	Yes	Yes	No	No	No
AS04	36	Female	El Salvador	Secondary	Unemployed	11 months	Yes	Yes	Yes	No	No	No
AS05	26	Female	Nicaragua	University	Unemployed	7 months	Yes	Yes	Yes	No	No	No
AS06	30	Male	Honduras	Secondary	Formal	1 year	Yes	Yes	Yes	No	No	No
AS07	37	Male	Mexico	Primary	Formal	6 months	Yes	Yes	No	N/A	No	No
AS08	25	Male	Mexico	Primary	Formal	5 months	Yes	Yes	No	N/A	No	N/A
AS09	36	Female	Honduras	None	Unemployed	6 months	Yes	Yes	Yes	No	Yes	No
AS10	36	Female	Mexico	Secondary	Informal	6 months	Yes	Yes	No	N/A	No	No
AS11	25	Male	Mexico	Primary	Unemployed	3 months	Yes	Yes	No	N/A	No	No
AS12	52	Female	Mexico	Primary	Unemployed	5 months	Yes	Yes	No	N/A	No	No
AS13	30	Male	El Salvador	University	Unemployed	1 year	Yes	Yes	Yes	No	No	No
AS14	43	Male	Honduras	Secondary	Unemployed	7 months	No	Yes	Yes	No	No	No
AS15	35	Male	Honduras	Primary	Unemployed	1 year	Yes	Yes	Yes	No	Yes	No
AS16	50	Male	Honduras	Secondary	Formal	9 months	No	Yes	Yes	No	No	No
AS17	28	Male	Cuba	Secondary	Formal	9 months	Yes	No	Yes	No	No	No
AS18	27	Female	Bolivia	University	Unemployed	8 months	Yes	Yes	Yes	No	No	Yes
AS19	26	Female	Honduras	Secondary	Unemployed	8 months	Yes	Yes	Yes	No	No	No
AS20	52	Female	Guatemala	Primary	Unemployed	6 months	No	Yes	Yes	No	No	No
Totals:	(24–52 range); average 34.4 years	55% female	7 countries	85% secondary or less	65% unemployed	(3–12 months range); average 9 months	85%	95%	75%	0%	10%	5%

This table describes the demographic features of asylum seeker participants from the Matamoros, Mexico asylum seeker camp. Interviews were conducted in August 2020.

**Table 2.** Demographics of Health Care Professionals Working with Asylum Seekers in Matamoros, Mexico

	Age	Gender	Education	Role	Asylum seeker	Direct health care delivery	Time working with population	Interdisciplinary work	Worked previously with vulnerable populations
HP01	60	Female	Master's in education	Health care services delivery	No	No	2 years	Yes	Yes
HP02	30	Male	University	Emergency services delivery, project manager	No	Yes	1 month	Yes	Yes
HP03	46	Female	University	Pharmacist	Yes	Yes	1 year	Yes	No
HP04	66	Female	Master's	Nurse	No	Yes	8 months	No	Yes
HP05	56	Male	University	Nurse	Yes	Yes	6 months	Yes	Yes
HP06	32	Male	Postgraduate degree	Physician	No	Yes	0.5 month	Yes	Yes
HP07	67	Female	Master's in theological studies	Health care services delivery	No	No	2 years	Yes	Yes
HP08	34	Male	Postgraduate degree	Physician	Yes	Yes	5 months	Yes	Yes
HP09	36	Female	University	Translator	Yes	Yes	7 months	Yes	No
HP10	30	Male	Postgraduate degree	Physician	No	Yes	6 months	Yes	No
Totals	(30–67 range); average 45.7 years	50% female	100% advanced degrees		40%	70%	(0.5–24 months range); 9 months average	90%	70%

This table describes the demographic features of health care professional participants caring for asylum seekers in the Matamoros, Mexico asylum seeker camp. Interviews were conducted in August 2020.

Our sample was considered representative of the population of asylum seekers in the camp. Among ∼2000 total residents at the time of interviews, ages ranged from newborn to late 60s, with mean ages of those in the camp in the late 30s. Almost all residents came from Latin America, with Honduras, Nicaragua, and Guatemala being the top three countries represented. Detailed statistics on pre-existing conditions of the population were not available. Roughly 30 aid workers from various NGOs were involved in health care services delivery.

### Organizational response to COVID-19

Organizations adopted numerous policies to reduce anticipated effects of COVID-19. Health care professionals reported that the preexisting Dignity Village structure allowed for a well-coordinated COVID-19 response. However, interventions varied in effectiveness and acceptance among asylum seekers. Positively viewed policies included improved water, sanitation and hygiene (WASH), contracting sanitation crews, supplying masks and disinfectants, and COVID-19 information campaigns. Negatively viewed policies were obligatory temperature checks and creation of a field hospital to isolate COVID-19 positive patients, the latter of which was perceived by asylum seekers as unnecessary and an exploitative tactic for fundraising. Other negatively perceived policies were construction of a camp perimeter fence and compulsory quarantine after a positive COVID-19 antibody test, as these policies lacked community engagement and threatened the autonomy of camp residents. The most negatively perceived policy was compulsory quarantine in the field hospital after a positive COVID-19 test (Table 3). Designed to quickly isolate positive patients, this policy was enacted without community education on reasons for quarantine or how long patients could be isolated. Beyond infringing on autonomy, this policy threatened to separate infected parents from their children. Due to fear of compulsory quarantine, asylum seekers reported being unwilling to be tested for COVID-19, or to access health care services even when they presented with COVID-19 symptoms:

**Table 3.** Policies Implemented to Address COVID-19 Disease Reduction and Risk Mitigation and Response from Asylum Seekers in Matamoros, Mexico

Policy	Goal	Asylum seeker sentiment	Example quote or explanation
Information campaigns	Educate asylum seekers on the signs, symptoms, and disease reduction strategies of COVID-19 and other preventive measures.	Positive	HP08: “We always meet with the leaders and explain in detail all that we could do to avoid a pandemic in the camp: hand washing, using hand sanitizer, obligatory mask wearing, restricted entries. Thanks to these information sessions, we haven't had any problem with COVID.” -Physician and asylum seeker, Cuba
Increased hand hygiene stations	Installation of water tubs with soap at various camp locations to facilitate hand washing and improve hand hygiene access	Positive	HP02: “Our WASH team has done an amazing job putting handwashing stations and making sure everyone knows they are safe to access. The washer structure here is impressive.” -Emergency services project manager, the United States
Increased wash stations	Increased clean water washing stations for clothes and dishes	Positive	
Increased bathroom number	Increased number of porta-potties for camp residents, to comply with international WASH guidelines	Positive	HP06: “Sanitation is a big public health issue. It's been great to see hand washing stations, showers, porta-potties, clothes and laundry washing stations.” -Physician, the United States
Sanitation crews	Contracted crews of asylum seeker employees to conduct twice daily sanitations of portable toilets and communal sanitation areas to reduce disease transmission	Positive	This policy was appreciated by asylum seekers, as it increased sanitation measures and provided a stable income for a group of camp resident asylum seekers.
Access to soap and other disinfectants	Provided supplies of soap, hand sanitizer, bleach and other hygiene and cleaning supplies.	Positive	AS03: “There's always soap to wash hands and masks and hand sanitizer. You go to the bathroom and there's a huge bottle of sanitizer or soap that you can use.” -Asylum seeker, El Salvador
Mask distribution and sewing	Distributed masks to asylum seekers to reduce COVID-19 infection, and have established a cooperative where asylum seekers can make and sell masks	Positive	AS01: “At first they gave us a cleaning kit with little hand sanitizers and other supplies. There are a ton of masks. I even have a collection of different designs; it's basically an accessory now.” -Asylum seeker, El Salvador
Positioning medical personnel outside camp	With reduced entry restrictions for the camp due to COVID-19, non-residents could no longer access NGO health care services. To care for asylum seekers living outside of the camp, organizations established a satellite tent clinic to overcome this restricted access.	Positive	HP05: “Since camp visits are restricted from COVID, all the asylum seekers living outside the camp cannot access the medical team in the camp. That's a huge barrier for many migratory asylum seekers. So we took an alternative approach to set up a medical post outside the camp to take care of everyone. We've looked for solutions to overcome these barriers and had great results while caring for everyone.” -Nurse and asylum seeker, Cuba
Suspended community health activities	Suspended community health workshops and other large community gatherings to reduce transmission risk. Although this was disappointing, residents understood the need for this policy.	Indifferent	HP06: “If we want to do dental health or preventive health education, we can't do it in large groups. We have to do it tent to tent or in small groups with little kids. In terms of daily rounds, I still see the families in their tents whenever.” -Physician, the United States
Masking when in the food reception line	Asylum seekers were required to wear masks and practice social distancing to reduce disease transmission when forming lines to collect food and other supplies.	Indifferent	Asylum seekers understood the importance of this policy and were generally willing to comply with mask wearing when they gathered to receive food and other supplies. However, many reported that social distancing in these settings was infeasible.
Fence construction and restriction of camp entry	The INM constructed a fence around the camp perimeter to control camp migration to reduce disease spread from unknown people.	Negative	Asylum seekers perceived the construction of a fence around the camp perimeter as a threat to personal safety, as they lacked escape options in emergency situations. Many preferred increased restrictions on camp entry to constructing a permanent barrier.
Temperature checks	Temperature checks with infrared guns were performed when entering or exiting the camp, before a medical consultation, or randomly during tent wellness check-ups, to identify residents with fever and potential COVID-19 cases.	Negative	AS07: “When you leave, they take your temperature and that's why we don't leave anymore. Every time they took my temperature, I had a really bad headache. Doing it always hurts me and we heard it can ruin your brain. So I don't leave the camp anymore because I don't want them checking my temperature.” -Asylum seeker, Mexico
Required COVID-19 antibody testing	Patients presenting to clinic and those with symptoms were tested to identify COVID-19 antibody cases. Health care professionals were unsure why they had COVID-19 antibody tests, but no viral PCR testing capabilities.	Indifferent	HP02: “It did create confusion when we were testing people. At one point we were testing everyone to get the data set, but a lot of people didn't want to come back after that. So we stopped obligatory testing. But if people want the test, we will offer it to them.” -Emergency services project manager, the United States
Field hospital construction	Constructed a 12-bed, secure health care facility to isolate and care for COVID-19 patients, provide quality care in a controlled setting and reduce disease transmission to uninfected residents.	Negative	AS08: “We understand that the organizations get a lot of money from us. They made a hospital and said that COVID is worse than it was so they could put people with any symptoms into isolation. We heard rumors that they were getting money to take care of the sick when in reality no one was infected.” -Asylum seeker, Mexico
Compulsory quarantine	Isolated patients who tested positively for COVID-19 antibodies, independent of symptoms, to reduce potential disease transmission to uninfected community members.	Negative	AS09: “The doctors only think about COVID. That's why they believe anyone with a cough has COVID, and they sent everyone to be isolated for prevention. They quarantined one guy away from his kids, and just left them alone for days. I said there's no way I'm going to the clinic, because they can't separate me from my little girls because they are my life.” -Asylum seeker, Honduras
Outsource food procurement	Organizations providing food shifted services from daily delivery by U.S. volunteers to procurement at local Matamoros restaurants, to reduce border travel and potential transmission by volunteer groups.	Negative	AS10: “The food is terrible, and I try not to give it to my daughters. It makes us sick. Before, the Americans would bring us food, which was clean and homemade, but now they cannot because of COVID. It is made in local restaurants, but we are seeing videos of unsanitary conditions. It was common to find a cockroach or fly in your food.” -Asylum seeker, Mexico
Suspension, reduction of activities by non-health care organization activities	Non-medical organizations shifted their service delivery model to work remotely, or suspended activities to lessen frequency of border crossings to reduce transmission.	Negative	AS16: “Since COVID we have felt a little forgotten because less volunteers are visiting. It's different from before when they would come, share and interact with us. So suddenly we feel a bit abandoned, but we know this is due to the pandemic and that spiritually they are with us.” -Asylum seeker, Honduras

This table describes the policies implemented to prepare for and in response to the COVID-19 pandemic in the Matamoros, Mexico asylum seeker camp, as well as asylum seekers participants' sentiments toward these policies. Quotes and explanations from health care professionals and asylum seeker participants are included in the rightmost column to demonstrate qualitative perceptions and explain nuances toward these policies.

INM, Instituto Nacional de Migración; NGO, nongovernmental organization; WASH, water, sanitation and hygiene.

*AS18:* “I had all the symptoms. I was in bad shape but when the organizations came to visit, I told them I felt fine because I didn't want to be isolated. Many friends had positive tests without symptoms, and they were all quarantined. I think it was a lie because everyone felt fine, and if we did have COVID we'd all be affected.”

-*Asylum seeker, Bolivia*

Country leaders, who were asylum seeker representatives elected to interface with the organizations (Fig. 1), objected to this policy, which, within 10 weeks, was changed to self-isolation in patients' tents. Health care professionals acknowledged that adverse effects from this policy were exacerbated by power dynamics between U.S. aid workers and asylum seekers. They expressed concern that distrust of organizations and fear of testing could be long-lasting and lead to underreporting of COVID-19 cases and decreased utilization of health care services by asylum seekers.

### Challenges throughout the COVID-19 pandemic

Participants reported challenges due to the COVID-19 pandemic, including mental health effects, health care access barriers and reduced access to the social determinants of health. Asylum seekers expressed mental health challenges, including fear of COVID-19 disease for themselves and their children (65%). Most qualified that this concern paled in comparison to other anxieties, including a lack of safety, surviving inhumane conditions, and aggravated emotional turmoil over suspension of asylum cases due to COVID-19:

*AS06:* “COVID has affected people's mentality because they are frustrated. Apart from closing the border, this virus is harming our asylum process and causing anxiety. People are mentally deteriorating and complaining about high temperatures and headaches. It's not physical because no one is infected, but mental.”

-Asylum seeker, Honduras

The physical effects of COVID-19 were much milder. Only one participant had tested positive for the coronavirus, whereas three asylum seeker participants knew residents who tested positive. No participants knew of cases of COVID-19 hospitalization or death (Table 4). Health care professionals expressed surprise and relief at the low levels of morbidity, with no patients requiring advanced care. They hypothesized that most infected people were asymptomatic, avoided testing, or attributed symptoms to other infections. Participants mentioned that COVID-19 exacerbated existing health care access barriers for asylum seekers, including safety accessing Matamoros hospitals and transportation, accessing identifying documents, discrimination, medication access, and payment ability:

**Table 4.** Public Health Compliance, Knowledge Levels, and Perspectives of Asylum Seekers Regarding Coronavirus Disease 2019 in Matamoros, Mexico (*n*=20)

Question	Number	Frequency
COVID-19 knowledge		
Has access to sources of information about COVID-19	19	95%
Has received information about COVID-19	19	95%
Demonstrated accurate knowledge of COVID-19	17	85%
Demonstrated inaccurate knowledge of COVID-19	10	50%
COVID-19 perceptions		
Tested positive for COVID-19	1	5%
Suspects may have been positive for COVID-19 at some point	1	5%
Knows another camp resident who was COVID-19 positive	3	15%
Questions COVID-19 being real or the seriousness of infection	7	35%
Fear of COVID-19 infection	13	65%
COVID-19 is a pressing concern to participant's life	12	60%
COVID-19 is the biggest concern in participant's life	1	5%
Belief in special protections against COVID-19^a^	11	55%
Faith in God, divine power	10	50%
Strong immune system	2	10%
Has access to masks	20	100%
Has access to disinfectants (soap, bleach)	20	100%
Has access to clean water	8	40%
COVID-19 has exacerbated existing health care access barriers	16	80%
COVID-19 has negatively affected access to the social determinants of health	16	80%
COVID-19 has negatively affected public health situation	10	50%
COVID-19 disease reduction compliance		
Attempts to comply with COVID-19 disease reduction strategies	19	95%
Believes following COVID-19 disease reduction strategies are important	18	90%
Believes it is possible to follow disease reduction strategies in the camp	11	55%
Wears a mask	19	95%
Outside camp	18	90%
Inside camp	3	15%
Shares mask with others	3	15%
Practices social distancing	18	90%
Outside camp	18	90%
Inside camp	0	0%
Practices hand hygiene	17	85%

This table describes the perceptions and behaviors of asylum seekers in Matamoros, Mexico regarding COVID-19 disease reduction strategies, as well as access to supplies to comply with public health measures. Disease reduction strategies included mask wearing, social distancing, disinfecting personal belongings, and hand hygiene.

^a^ One participant mentioned special protection from both God and increased immunity.

COVID-19, coronavirus disease 2019.

*HP09:* “COVID has a huge impact on healthcare access here. People feared becoming infected if they travel to the hospital or isolated if they were positive. This fear, combined with other barriers like discrimination, economic, and distrust of institutions created a lot of problems.”

-Medical translator and asylum seeker, Peru

Our sample highlighted that COVID-19 affected the social determinants of health in multiple ways, including food access, personal safety, and employment. Though nutritious food was previously delivered by aid organizations, the shift to local procurement resulted in unsanitary conditions, which increased gastrointestinal diseases. With personal safety, participants reported feeling trapped within the camp, as traveling outside may result in increased chance of COVID-19 disease. Our sample worried that their escape options in cases of violence within the camp were limited. Participants reported employment loss due to COVID-19, as businesses in Matamoros were down-sized. The loss of income made asylum seekers less likely to find stable housing or afford legal or translation services.

### COVID-19 perceptions and behaviors of asylum seekers

Participants reported receiving information about COVID-19 from Dignity Village, personal contacts, and the Internet through mobile devices (95%). Many demonstrated knowledge of COVID-19, including understanding of transmission and disease reduction strategies. However, there were discrepancies in knowledge accuracy, with 50% making inaccurate claims:

*AS20:* “We disinfect all the food with bleach. Vegetables, meat, fruit, whatever we're going to eat is cleaned with bleach because it can eliminate any bacteria that could make someone sick.”

-Asylum seeker, Guatemala

Asylum seekers were unconcerned about COVID-19 within the camp. Due to the absence of symptomatic cases, nearly every asylum seeker believed that COVID-19 would not affect residents, and one-third questioned the seriousness of the disease (35%). There was discrepancy between participants' theoretical knowledge in describing disease reduction strategies, and their practical belief that they were safe from coronavirus complications. Participants paradoxically viewed the camp as safe from infection, and worried about increasing exposure risk traveling into Matamoros city. This perception influenced compliance with disease reduction strategies, which were practiced regularly outside the camp, but rarely within. Participants viewed mask wearing inside the camp as a sign of potentially being COVID-19 positive, since most mask-wearers were aid workers from the outside. When leaving the camp or visiting in-camp clinics, asylum seekers judiciously wore masks and practiced social distancing. Compliance with disease reduction strategies was motivated by protecting others for the common good:

*AS16:* “I don't wear my mask in the camp because there isn't any [COVID-19] here. I do take precautions when I leave because outside it is unsafe. Here we are responsible as a community, and I should always be careful because not taking precautions could put my neighbor, family or community at risk.”

-Asylum seeker, Honduras

There was also discrepancy between participants' understanding and ability to implement disease reduction strategies. Though some questioned the seriousness of the disease, nearly all participants expressed that they understood the importance of masking, social distancing, and hand hygiene to reduce disease transmission in theory. However, social distancing was unfeasible within the camp, as tents were within inches, and activities of daily living were performed in close quarters. Participants reported that masking and hand hygiene were feasible and affirmed that they had access to masks, disinfectants, soaps, and hand sanitizer. There was discrepancy about clean water access for washing, cooking, and drinking. Many asylum seekers believed that they had special protective measures to prevent COVID-19 disease (55%). These included a strong immune system from living in inhumane conditions, as one Guatemalan woman expressed that she cannot contract COVID-19 after having survived natural disasters, other infections, and living among disease vectors, including rats and mosquitos. Another was the belief that God was protecting participants from COVID-19. Some used their faith to manage anxieties around infection, whereas others believed that it made them immune:

*AS11:* “I don't believe in COVID. I believe in God. You can try to prevent it with masks, but this only helps a little. Only God can care for all of us, but I still wear a mask to take care of others.”

-Asylum seeker, Mexico

Health care providers shared a different view of COVID-19. Most believed that it had infected many residents but were surprised there were no cases requiring hospitalization. Some postulated that the open-air conditions, WASH infrastructure, or young population demographics reduced complication rates.

## Discussion

This study provides findings on the effects, challenges, and perceptions of COVID-19 for asylum seekers at the U.S.-Mexico border. Our results help fill the qualitative data gap on COVID-19 among asylum seekers and can inform policy to address these challenges. Our findings show that among a diverse population of asylum seekers with varied perceptions and public health compliance, COVID-19 created unique challenges for asylum seekers and their health care providers. Although participants reported mental health effects including increased anxieties, surprisingly few noted adverse physical outcomes. There were no hospitalizations, and those with relevant histories denied infection. Compared with higher rates of morbidity and mortality among other vulnerable groups in congregate settings,^26,27^ this setting could be a helpful COVID-19 epidemiological case study. It would be useful in future research to explore factors that might have contributed to the low observed morbidity and mortality in this population at the time of this study.

Challenges from COVID-19 included reduced access to health care services and the social determinants of health. The social determinants of health that were especially noted by our sample included decreased access to nutritious food, personal safety in instances of violence, employment opportunities in Matamoros, and education for children and teenagers. COVID-19 decreased access to nutrition, employment, asylum claims, and Mexican health facilities due to financial, transport and discrimination barriers. These challenges were perceived to affect mental and physical health of asylum seekers more than COVID-19. Similarly, research in an Iraqi refugee camp demonstrated increased post traumatic stress disorder rates and mental health effects when COVID-19 influenced residents' social determinants of health.^28^

Asylum seekers demonstrated varied levels of COVID-19 knowledge and compliance to disease reduction strategies. Nearly every participant expressed the importance of social distancing, hand hygiene, and mask wearing outside the camp, whereas few did so within the camp due to infeasibility and their own risk perceptions. There are opportunities to develop creative COVID-19 management strategies that are more acceptable in such settings. Useful interventions among the Rohingya have included training for religious leaders, electronic billboards distributing COVID-19 information, and digital health booths for physician consults.^9,29^ Most asylum seekers did not believe they were at-risk for COVID-19 disease in the camp, with reasons varying from adherence to public health measures, to special defenses including strong immunity and protection from God. One study found that religiosity correlated to decreased adherence of COVID-19 mitigation guidelines,^30^ supporting our findings and suggesting that religious beliefs could play a role in COVID-19 perceptions and education. No other research discussed a belief of protection from COVID-19 due to God, marking a novel finding.

Asylum seekers reported COVID-19 information access, but knowledge levels were inconsistent. These discrepancies could be due to contradictory information from various sources, as COVID-19 understanding is constantly changing. Studies in Bangladesh demonstrated Internet access as a key factor to information access for refugees.^9,29^ As Internet usage becomes more prevalent in these settings,^31–33^ there may be increasing need to validate accurate health information for asylum seekers. Knowledge gaps are important to address, as COVID-19 knowledge is a key factor in quarantine adherence.^34^ Though this study did not measure quantitative knowledge scores for COVID-19, future research on this topic could determine opportunities for public health education.

There was discrepancy between health care providers and asylum seeker perceptions on the seriousness of COVID-19, which created tensions after the implementation of new COVID-19 policies. Resistance surfaced when policies curtailed autonomy or were perceived as unnecessary. The construction of a field hospital and compulsory quarantine resulted in distrust, which could last beyond the pandemic. Although these were reasonable policies to reduce adverse outcomes, the negative effects may have been mitigated by focusing on community engagement and including asylum seekers in protocol development to make measures more acceptable.

Participants believed there was underreporting of COVID-19, due to lack of viral testing, misunderstanding of testing importance, belief in special protections, and fear of compulsory quarantine. A study among Rohingya refugees noted participants' fear that COVID-19 carriers would be refused treatment, reflecting our finding that fear of a positive COVID-19 test led to underreporting.^28^ Future studies could focus on implementing policies that encourage testing, while not threatening patients' access to care.

There were expected findings not mentioned by participants. None reported a lack of personal protective equipment, a testament to organizations' well-coordinated services. In other refugee camps, research demonstrated a lack of Internet, inadequate sanitation, and misinformation about punishments for COVID-19-positive cases to be issues.^9,35^ These challenges were not mentioned by our sample and could be explained due to higher resources and Internet access, organizations' efforts to fortify WASH infrastructure, and collaborations between the NGOs and asylum seekers.

### Limitations

Though a qualitative approach was appropriate for an exploratory study, quantitative data on COVID-19 knowledge scores and services utilization could provide important results. Health care staff from Médicos Sin Fronteras (MSF) and Instituto Nacional de Migración (INM), two additional assistance organizations, were not interviewed. Incorporating MSF providers may yield more mental health information, as they manage these services. Finally, our sample was limited to a specific population in one location. Although these findings are not generalizable to all asylum seekers along the U.S.-Mexico border, this methodology could help design research in similar settings.

### Health equity implications

This study revealed lessons in addressing COVID-19 at the U.S.-Mexico border among asylum seekers and could have implications for managing disease outbreaks in other refugee settings. Implementing new policies that threatened patient autonomy without community engagement caused distrust and diminished health care seeking. Many traditional disease reduction strategies, including social distancing and mask wearing, were not feasible or acceptable to asylum seekers, suggesting the need for creative new measures with a community-based design. By operating as a collaborative network, organizations quickly implemented measures to address COVID-19, and communicated with asylum leaders for rapid adjustment according to community needs. Finally, though asylum seekers expressed fear of COVID-19, access to legal services and the social determinants of health remained top priorities. Although addressing disease outbreaks in these settings is crucial, it should be done within a complete framework that addresses preexisting health care challenges and reinforces the social determinants of health.

## Supplementary Material

Supplemental data

Supplemental data

## Abbreviations Used

COVID-19: coronavirus disease 2019
INM: Instituto Nacional de Migración
MPP: Migrant Protection Protocols
MSF: Médicos Sin Fronteras
NGO: non-governmental organization
WASH: water, sanitation and hygiene
