# Supplementary material for: Challenges and Effects of the COVID-19 Pandemic on Asylum Seeker Health at the U.S.-Mexico Border
Source: Health Equity. 2021 Apr 13;5(1):169–80. doi: 10.1089/heq.2020.0110 (PMC8080921; doi:10.1089/heq.2020.0110)
Supplement: Supplemental data [file Supp_AppenS1.docx]

**ENTREVISTA SEMI-ESTRUCTURADA, Solicitantes de asilo**

¿Ha leído el consentimiento informado? (si/no)

¿Tiene alguna pregunta en este momento? (si/no)

¿Le gustaría participar? (si/no)

1. **Aspectos demográficos**
   1. ¿Cuántos años cumplidos tiene?
   2. ¿Es Usted hombre o mujer?
   3. ¿Dónde nació?
   4. ¿De dónde es usted?
   5. ¿Cuál es su nivel más alto de formación (educación)?
      1. Educación primaria
      2. Secundaria
      3. Técnica
      4. Profesional Universitario
      5. Maestría
      6. Doctorado
   6. ¿Usted tiene empleo?
      1. Si: ¿En que trabaja?
      2. No: ¿le gustaría trabajar si tuviera la oportunidad?
      3. ¿Cómo gana un ingreso usted?
   7. ¿En el pasado, en que ha trabajado?
   8. Verificación
      1. ¿Cuánto tiempo usted lleva en el campamento?
      2. ¿Usted vive de tiempo completo en el campamento?
         1. No: ¿dónde vive actualmente?
      3. ¿Cómo llegó a este campamento?
         1. ¿Usted llegó con una familia o solo/a?
         2. ¿Tiene hijos?
      4. ¿Usted ha aplicado para asilo en los EEUU?
         1. ¿Ha recibido una respuesta?
         2. ¿Has pensado regresar a su país o hogar?
         3. ¿Has aplicado para asilo en otro país?
2. **Determinar las posibles barreras a los servicios de salud que enfrentan los solicitantes de asilo.**
   1. ¿Me podría explicar el proceso de obtener servicios de salud cuando usted se enferma?
   2. ¿Cuáles son las principales barreras a sistemas de salud que usted percibe para usted y otros solicitantes de asilo?
   3. ¿Cuál es su mayor duda o queja sobre el sistema de salud?
   4. ¿Usted tiene acceso a los servicios de salud en Matamoros?
   5. ¿Tiene un tipo de aseguramiento social?
      1. Si se enferma de una enfermedad grave o una emergencia, ¿cómo obtiene usted ayuda profesional?
   6. Fuera de temas de salud, ¿qué aspectos percibe como los más importantes para los solicitantes de asilo?
   7. ¿Qué opinas de su experiencia con el sistema de salud en este momento?
      1. ¿Qué opinas de su acceso a servicios de salud actualmente?
   8. En un mundo / sistema ideal, como se ve su acceso al sistema de salud en el futuro?
3. **COVID**
   1. ¿Usted ha recibido información sobre la infección de COVID / coronavirus?
   2. ¿Cómo le ha afectado la situación de COVID?
      1. ¿Hay más o menos servicios / acceso a servicios por el COVID?
         1. ¿Cómo te ha afectado en términos de acceso a servicios de salud?
      2. ¿Usted tema por contagiarse del COVID?
      3. ¿Es posible hacer medidas de control y prevención en el campamento por el COVID?
      4. ¿Usted tiene acceso a jabón? ¿O agua limpia?
      5. ¿Usted se ha enfermado del COVID?
   3. ¿Tienes la habilidad de seguir con las precauciones para reducir la transmisión de COVID? (mascarilla, distancia social, etc.?)
      1. ¿Usted lleva una mascarilla / cubreboca?
         1. ¿Qué tan frecuente llevas tu mascarilla?
         2. ¿Comparte tu mascarilla con otras personas de su familia o comunidad?
      2. ¿Cuantas veces al día lavas las manos?
4. **Determinar la situación de salud del participante:**
   1. ¿Usted tiene una enfermedad crónica?
      1. ¿Cómo obtiene los medicamentos necesarios para controlar esta condición?
   2. ¿Qué tipo de servicios de salud le hace falta?
   3. ¿Usted tiene acceso a servicios de salud mental?
      1. ¿Usted le gustaría consultar a un psicólogo u otro profesional de salud mental?
   4. ¿Aprecia el trabajo de los médicos y organizaciones que provienen asistencia al campamento?
   5. ¿Usted tiene confianza que los profesionales de salud le quieren ayudar?
      1. ¿Puede hablar honestamente con los profesionales de salud y trabajadores de los ONGs del campamento sobre su estado y necesidades de salud?
5. **Determinar el estado del ETCR y las experiencias brindando salud en esa área.**
   1. ¿Cuáles son los problemas de salud pública en el campamento?
      1. ¿Cómo se puede mejorar la situación de salud pública en el campamento?
      2. ¿Cómo ha afectado la situación de salud pública la enfermedad COVID?
6. ¿Qué razones tuvo para tomar la decisión de inmigrar a los EEUU?
7. **Brindar recomendaciones tangibles sobre cómo mejorar el acceso a la atención médica de los solicitantes de asilo.**
   1. ¿Qué se puede hacer para ayudar a mejorar la asistencia médica de los solicitantes de asilo?
   2. ¿Basados en su experiencia en la atención a los solicitantes de asilo, que recomendaciones haría al gobierno mexicano, Naciones Unidas, y las ONGs para intervenciones tangibles para mejorar su acceso a servicios de salud?
