# Supplementary material for: Challenges and Effects of the COVID-19 Pandemic on Asylum Seeker Health at the U.S.-Mexico Border
Source: Health Equity. 2021 Apr 13;5(1):169–80. doi: 10.1089/heq.2020.0110 (PMC8080921; doi:10.1089/heq.2020.0110)
Supplement: Supplemental data [file Supp_AppenS2.docx]

**SEMI-STRUCTURED INTERVIEW, asylum seekers and healthcare professionals**

Have you read the informed consent? (yes/no)

Do you have a question at this time? (yes/no)

Would you like to participate? (yes/no)

1. **Demographics**
   1. How old are you?
   2. What is your gender?
   3. Where were you born?
   4. Where are you from?
   5. What is your highest level of education?
      1. Primary
      2. Secondary
      3. University degree
      4. Master’s
      5. Doctorate
   6. Are you employed?
      1. If yes, what is your work?
      2. If no, would you like to work if you had the opportunity?
      3. How do you earn an income?
   7. In the past, in what areas have you worked?
   8. Verficiation
      1. How long have you lived in the Matamoros camp?
      2. Do you live full-time in the camp?
         1. If no, where do you live now?
      3. How did you arrive to this camp?
         1. Did you travel with family or alone?
         2. Do you have children?
      4. Have you applied for asylum in the United States?
         1. Have you received a response?
         2. Have you thought about returning to your home country?
         3. Have you applied for asylum in another country?

1. **Determine possible barriers to healthcare services that face asylum seekers**
   1. Could you explain the process of obtaining healthcare services when you get sick?
   2. What are the main barriers to healthcare services that you perceive for yourself and other asylum seekers?
   3. What is your biggest complaint or doubt about the health system?
   4. Do you have access to health services in Matamoros?
   5. Do you have any type of insurance?
      1. If you were to get sick with a serious condition or emergency, how would you obtain professional help?
   6. Outside of health, what aspects of life do you perceive as the most important for asylum seekers?
   7. What is your opinion of your experience with the healthcare system at this time?
      1. What do you think of your access to healthcare services currently?
   8. In a perfect world or system that you could design, how would you see healthcare services for asylum seekers working?
2. **COVID**
   1. Have you received information about COVID / coronavirus?
   2. How has COVID affected you?
      1. Are there more or less health services because of COVID?
         1. How has it affected you in terms of healthcare services?
      2. Are you afraid of becoming infected with COVID?
      3. Is it possible to implement COVID prevention measures in the camp?
      4. Do you have access to soap, or clean water?
      5. Have you gotten sick because of COVID?
   3. Do you have the ability to follow cautions to reduce COVID transmission (masking, social distancing, etc.?)
      1. Do you wear a mask?
         1. How often do you wear your mask?
         2. Do you share your mask with other people from your community or family?
      2. How many times per day do you wash your hands?

1. **Determine the health situation of each participant**
   1. Do you have a chronic disease?
      1. How do you obtain necessary medications to manage this condition?
   2. What type of health services are you missing?
   3. Do you have access to mental health services?
      1. Would you like to consult a psychologist or other mental health professional?
   4. Do you appreciate the work of the doctors and organizations that provide aid to the camp?
   5. Do you trust that the healthcare professionals want to help you?
      1. Can you speak honestly with the healthcare professionals and NGO workers of the camp about your healthcare status and needs?
2. **Determine the state of the camp and experiences delivering healthcare in this area**
   1. What are the public health problems in the camp?
      1. How could one improve the public health situation in the camp?
      2. How has COVID affected the public health situation?
3. What reasons did you make the decision to immigrate to the United States?
4. **Offer tangible recommendations about how to improve medication attention for healthcare attention for asylum seekers**
   1. What could be done to help better the medical aid of asylum seekers?
   2. Based on your experiences with this population, what recommendations would you give to the Mexican government, United Nations, and NGOs for tangible interventions to improve access to healthcare services?
